# Posters

**DOI:** 10.1038/sj.bjc.6601938

**Published:** 2004-06-22


**ROLE OF GLUCOSE TRANSPORTER 1 IN TUMOUR GROWTH AND RESPONSE TO THERAPY**

SJ Brittain-Dissont , KJ Williams , RE Airley , F Sheppard , A Evans , A Carruthers , IJ Stratford
United Kingdom, Experimental Oncology, Department of Pharmacy and Pharmaceutical Sciences, University of Manchester, Manchester

Glut-1 is a facilitative transporter of D-glucose that assists in regulation of glucose turnover in mammalian cells. It exists as a plasma membrane-spanning protein that translocates from vesicular stores to the membrane in response to hypoxia. During chronic oxygen and glucose deprivation, HIF-1 initiates transcriptional *de novo synthesis* of Glut-1 to counteract the detrimental conditions.
We are carrying out a comprehensive study of hypoxia induced Glut-1 expression in a diverse panel of tumour cell lines. These cells show an increase in Glut-1 protein expression following 18 hours exposure to hypoxia (0.001% $O_2$). Our current experiments are aimed at determining how long the elevated Glut-1 levels persist for following the return of cells to a normoxic environment. This is to gain an understanding of whether Glut-1 expression in tumours will provide a reflection of chronic and/or acute hypoxia. To carry out these experiments samples of the re-oxygenated cells are removed at two hourly intervals, centrifuged to form pellets and formalin fixed prior to histological processing and stained with antibodies specific for Glut-1. Preliminary evidence in Hepa-1 cells indicates Glut-1 levels remain high for at least six hours after hypoxia exposure but return to basal levels after 24 hours. These experiments are being repeated in other cell lines. The effect of over expressing exogenous Glut-1 in tumour cell lines is also under investigation. We have inserted the cDNA for Glut-1into pEFIRES-P a constitutive mammalian expression vector developed by Hobbs *et al* (1998) for stable cell line generation and we are currently generating clones. These clonal cell lines will be used to investigate and validate glucose uptake into tumours and its effect on tumour growth. In addition to constitutively over-expressing Glut-1 we propose to utilize the tetracycline inducible-gene expression system. This should enable assessment and quantification of glucose uptake relative to the intensity of membrane-bound Glut-1.

### P92

**ADENOVIRUS-MEDIATED DELIVERY OF A HYPOXIA-REGULATED CARBOXYLESTERASE GENE FOR TUMOUR-DIRECTED ACTIVATION OF IRINOTECAN (CPT-11)**

T Matzow [1], PJ Flint [1], RL Cowen [2], KJ Williams [2], IJ Stratford [2], MP Saunders [1]
[1] Paterson Institute for Cancer Research, Manchester, United Kingdom, [2] School of Pharmacy and Pharmaceutical Sciences, University of Manchester, Manchester, United Kingdom

Irinotecan (CPT-11), a derivative of the topoisomerase I inhibitor camptothecin, is a chemotherapeutic agent currently used in treatment of colorectal, stomach and pancreatic cancers. Having poor activity on its own, carboxylesterase (CE) enzymes convert it into the much more potent compound SN-38. CEs are most abundant in the liver, where two distinct isotypes, hCE-1 and hCE-2, have been characterised, the latter being approximately 50-fold more efficient at CPT-11 conversion. A third human CE, hiCE, expressed predominantly in the small intestine, is almost identical to hCE-2. A rabbit liver CE, rCE, has been shown to convert CPT-11 to SN-38 with 100 to 1000- fold higher efficiently than hCE-1 *in vitro*, despite sharing >80% sequence homology. The conversion of CPT-11 to SN-38 by endogenous CE is not very efficient, and CE activity is often further reduced in tumour cells. However, due to dose-limiting toxicity (including severe diarrhoea), the administered dose of CPT-11 cannot be increased to fully compensate for the poor conversion rate.
Hypoxia is common in solid tumours due to inefficient and unstructured angiogenesis. The expression of many proteins, including VEGF, Epo and most glycolytic enzymes, is regulated by the hypoxia-inducable transcription factor HIF-1, which specifically binds to hypoxia responsive enhancer elements (HREs).
We have constructed recombinant adenovirus vectors carrying the gene for either hiCE or rCE, under the control of a HRE derived from the murine PGK-1 gene, in conjunction with a minimal SV40 promoter. We have demonstrated enhanced CE-expression combined with increased sensitivity to CPT-11 in response to hypoxia in cells pre-infected with these viruses *in vitro*, and are performing *in vivo* experiments with the vectors in tumour xenografts.
We hope therapeutic delivery of these vector constructs will allow a significant tumour-directed increase in CE expression, thus increasing the potency of CPT-11 administered at permissive doses.

### P93

**INHIBITION OF HYPOXIA-INDUCIBLE CA-IX MODULATES ANTICANCER DRUG UPTAKE AND TOXICITY**

CA Parker [1], M Jaffar [1], KJ Williams [1], PM Loadman [2], RM Phillips [2], IJ Stratford [2]
[1] University of Manchester, Manchester, United Kingdom, [2] University of Bradford, Bradford, United Kingdom

The presence of hypoxia within solid tumours is related to increased aggressiveness, resistance to therapies and consequently, poor prognosis. Carbonic anhydrase-IX (CA-IX) is a hypoxia-regulated enzyme, overexpressed in many types of human cancer. CA-IX is involved in pH homeostasis, contributing to extracellular acidification and tumourigenesis. Acidification of the extracellular milieu can impact upon cellular uptake of chemotherapeutic drugs by favouring weak acids (e.g. melphalan), but limiting access of weak bases (e.g. doxorubicin). Thus, the aim of this study was to determine whether alterations of CA-IX activity affected anticancer drug uptake and toxicity.
The addition of 50µM CA inhibitor, acetazolamide (AZM), enhanced doxorubicin toxicity up to 12 fold, but conversely reduced melphalan toxicity up to 10 fold in cell lines that highly expressed CA-IX under anoxic conditions (HT29 & MDA435 CA9/18). The changes in doxorubicin toxicity were reflected in increased passive uptake in anoxic HT29 and MDA435 CA9/18 cells. AZM limited depletion of GSH by melphalan in highly inducible cells under anoxic conditions, suggesting that melphalan uptake was reduced. AZM did not significantly alter toxicity or uptake in cells with low CA-IX activity (HCT116 & MDA435 EV1). 10-50µM AZM lowered the intracellular pH in HT29 & MDA435 CA9/18 cells under anoxic conditions. The effect of AZM on doxorubicin uptake and penetration is currently under investigation in spheroid and multicell layer models, using confocal microscopy and HPLC techniques.
CA-IX inhibition under anoxic conditions resulted in acidification of the intracellular environment and can enhance or diminish uptake/toxicity of specific anticancer agents. CA-IX has chemomodulatory properties and is an attractive target for cancer therapy.

### P94

**CORRELATIVE STUDY BETWEEN THE ANGIOGENESIS OF HCC AND ITS CLINICOPATHOLOGIC RESULTS**

Z Gao [1], DX Yu [2], B Wang [2]
[1] Department of Medical Biology, Weifang Medical University, Weifang, China, [2] Department of Radiology, Weifang, China

**Introduction** To study the relationship between the angiogenesis of hepatocellular carcinoma (HCC), HCC and its clinicopathologic results, and analyze the associations among microvessel density (MVD), P53 and vascular endothelial growth factor (VEGF).
**Material and Methods** The study group comprised 60 patients who underwent surgical treatment. Sections were stained with p53, VEGF and CD34immunohistochemically. The tumor size, capsule and edge of the lesions were recorded in surgery. The necrosis, hyaline-cell areas, fatty degeneration areas and grades of differentiation were recorded in pathology. The clinical and histopathological characteristics of the patients were compared with MVD, p53 and VEGF.
**Results** The MVD of HCC in the group without liver cirrhosis was lower than that in those of liver cirrhosis ($P < 0.05$). MVD did not correlate with hepatitis C, necrosis, fatty degeneration, histologic grade or capsule formation ($P > 0.05$). VEGF expression was significantly different between intrahepatic metastasis and non-metastasis (75% VS 43.18%, $P < 0.05$), vascular invasion and noninvasion (71.43% VS 41.03%, $P < 0.05$). Expression of VEGF was more frequently in HCC with hepatitis B ($P < 0.05$). No significant correlations were detected between VEGF expression and fatty degeneration, histologic grade, the size and liver cirrhosis. p53 expression showed a distinctly higher among these groups such as poorly and moderately differentiation to well differentiation(41.30% VS 7.14%, $P < 0.05$), vascular invasion to nonivasion (57.14% VS 20.51%, $P < 0.05$), intrahepatic metastasis to nonmetastasis (62.5% VS 22.73%, $P < 0.05$). No correlation was found betweeen hepatitis B and C status, the size and capsule formation. Significant correlation was also found among p53, VEGF and MVD.
**Conclusions** There is a statistically significant correlation between VEGF and P53 expression ($P < 0.05$). VEGF associated with P53 influence MVD. Both P53 and VEGF have a significantly improved efficacy in promoting tumor angiogenesis and metastasis in HCC.

© 2004 Cancer Research UK

### P94:1

**USE OF A NOVEL, HYPOXIA-RESPONSIVE DNA PROMOTER IN SALMONELLA TO TARGET GENE THERAPY TO HYPOXIC AREAS OF SOLID TUMOURS**

R Ryan [1], J Green [1], J Harmey [2], S Kehoe [2], C Lewis [3]
[1] United Kingdom, Molecular Biology and Biotechnology Dept., University of Sheffield, Sheffield, [2] United Kingdom, Dept. of Surgery, Royal College of Surgeons in Ireland, Education and Resecrch Centre, Beaumont Hospital, Dublin, [3] Ireland, Medical School, University of Sheffield, Sheffield

Multiple areas of hypoxia and anoxia are features of malignant tumours. Hypoxic tumour cells are non-proliferating, and therefore relatively refractory to radiotherapy and chemotherapy. This has prompted the development of alternative vectors to target therapeutic genes to these hypoxic tumour areas, including the use of attenuated strains of such bacteria as *Salmonella typhimurium.* The expression of therapeutic genes in normoxic tissues is a major problem but overcome if an additional level of targeting is included. This can be achieved by placing a therapeutic gene under the control of a hypoxia-inducible bacterial transcription factor called Fumarate and Nitrate reduction Regulator (FNR). Here, we show that an FNR-regulated promoter (FFp*melR*) activates expression of the reporter gene (*lacZ*) under hypoxic conditions in an attenuated *S. typhimurium* strain *in vitro*. Mutations in the FNR promoter improved anoxic induction of the *lacZ* reporter gene to around 625-fold relative to normoxia and decreased expression under normoxic conditions. This mutant FFp*melR*::*lacZ* construct in *S. typhimurium* was used to infect a human breast cancer cell line (T47D). *LacZ* was expressed when these infected cells were exposed to hypoxia (0.5% oxygen) but not normoxia (20.9% oxygen) for 16h *in vitro*. This *S. typhimurium* mutant was utilised to infiltrate T47D multicellular tumour spheroids. The bacteria infiltrated the spheroids but *lac*Z expression was restricted to the hypoxic areas of these tumour masses. In an *in vivo* experiment, *S. typhimurium* ($10^6$) transformed with either mutant FFp*melR*::alphastatin (alphastatin is a new anti-angiogenic peptide) or a null construct were injected (i.v.) into female BALB/c mice bearing 4T1 mammary tumours, and tumour volume monitored. The attenuated bacteria were well tolerated and tumour growth was halted in those injected with the FFp*melR*::alphastatin. We conclude that FNR-dependent promoters in *S. typhimurium* can be used to target therapeutic gene expression to hypoxic areas of murine tumours.

### P95

**PROTEOMIC CO-EXPRESSION PATTERNS OF CRITICAL NORMAL GENES ARE FOUND IN HUMAN CANCER CELL LINES BUT NOT IN NORMAL CELLS**

H. Warenius , l. Seabra , L. Kyritsi , M. Jones , C. Thomas , R. White , A. Howarth
University of Liverpool, Liverpool, United Kingdom

We have carried out proteomic profiling of 23 known proteins responsible for the control of cellular proliferation, differentiation, senescence and death in 20 human in vitro cancer cell lines covering a wide range of histologies and in primary cultures of normal keratinocytes and fibroblasts. The proteins include cyclins, cyclin dependent kinases, cyclin dependent kinase inhibitors, proto-oncogenes tumour suppressors and pro- and anti- apoptotic proteins in addition to normal controls. The proteomic profiles obtained are different for each individual cell line, consistent with the likelihood that each cancer cell may constitute a unique emergent system. However, certain patterns of close gene product interaction (with r values greater than 0.5 and p values greater than 0.05) are discernible. We suggest that these patterns of gene expression may reflect Critical Normal Gene Products whose co-expression is required to maintain Neostasis in inherently unstable cancer cells. These patterns of gene product co-expression are not dissimilar to appearances of scale free systems, where nodes of high interaction can provide the most vulnerable sites for disrupting the system. The proteomic database of the cell lines examined here is insufficiently large to demonstrate this formally. Nonetheless an understanding of these patterns of interaction may lead to different approaches towards identifying novel targets for new drug development.

### P96

**FUNCTIONAL ANALYSIS OF GENE EXPRESSION DIFFERENCES IN A MODEL OF COLORECTAL CANCER PROGRESSION**

E. Foran , F. Lennon , D.T. Croke , A.C. Long
United Kingdom, RCSI, Dublin

Our group has previously identified a set of genes to be differentially expressed between the premetastatic cell line SW480 and its metastatic counterpart, SW620 (1). The aim of this study was the identification of functional differences between SW480 and SW620. Migration and proliferation, two parameters associated with metastasis, were analysed using a phagokinetic track assay and an MTS assay respectively I
Two major areas of cell signalling were investigated –phosphorylation/dephosphorylation and the PI3-kinase pathway. Both pathways have previously been associated with cell migration and proliferation in epithelial cell lines. A number of interesting observations were made. First, a role exists for the protein phosphatases 1 and 2A in the control of proliferation in SW620. Also, inhibition of PI3-kinase by wortmannin in SW480 leads to the development of lamellipodia/pseudopodia in this cell line, but not in SW620, indicating that PI3-kinase may be involved in actin organisation in SW480. These data will contribute to current knowledge regarding SW480 and SW620 cell lines as a model of colorectal cancer progression.
In addition, our group has also determined that the L-plastin gene is up-regulated in SW620 with respect to SW480. SW480 cell lines stably expressing L-plastin were subsequently established (SW480-LPL) and analysis of these cell lines revealed a significantly higher rate of proliferation and lower rate of migration than the control cell line (SW480-pcDNA3.1). E-cadherin, an important tumour suppressor gene was lost from SW480-LPL cells. A link was elucidated between L-plastin induction and the down-regulation of E-cadherin, whose loss of expression is directly associated with the development of metastasis in several epithelial cancers. These data could be of importance in clarifying the mechanisms of colorectal cancer metastasis.

### P97

**ANALYSIS OF ABERRANT CPG-ISLAND METHYLATION IN STAGE III AND IV OVARIAN CANCER**

JM Teodoridis , L Braidwood , J Hall , PA Vasey , G Strathdee , R Brown
United States, University of Glasgow, Glasgow

Ovarian tumours are often diagnosed at late stages III or IV and are usually treated with surgery followed by platinum-based chemotherapy. Resistance to chemotherapy is a major cause of treatment failure. Inactivation of genes involved in cellular responses to DNA damaging agents, including genes involved in DNA repair, apoptotic signalling and cell cycle control have been identified as possible causes of tumour chemoresistance.. Transcriptional silencing of genes associated with aberrant methylation of CpG islands represents one possible molecular cause for chemoresistance and it has been suggested from small retrospective studies that aberrant DNA methylation can correlate with disease progression and overall survival in ovarian cancer patients (S. H. Wei et al., Clinical Cancer Research 8(7), p. 2246 (2002)). We set out to investigate if aberrant DNA methylation of genes involved in cellular responses to DNA damage can be used to predict tumour response to chemotherapy or patient survival. To achieve this, DNA from 98 stage III or IV ovarian tumours taken at presentation was isolated, bisulphite modified and analysed by methylation specific-PCR. CpG island methylation was seen in *apaf-1* (14.3%), *blu* (34.7%), *brca1* (19.4%), *casp8* (2%), *dapk* (2%), *gst*π (2%), *mgmt* (10.2%), *mlh1* (15.3%), *p14* (1%), *p16* (3.1%), *p21* (27.6%), *p73* (4.1%), *rassf1a* (38.8%), but not in *fancf*, *fas* or *survivin*. Methylation of at least one of these genes was observed in 80.6% of tumours. Thus, CpG island methylation is a frequent event in late stage ovarian tumours. Statistical analysis of associations between methylation and clinical outcome is currently underway.

© 2004 Cancer Research UK

### P98

### DETECTION OF GENETIC ALTERATIONS IN NON-SMALL CELL LUNG CANCER (NSCLC) USING INTER-SIMPLE SEQUENCE REPEAT PCR

DM Broomhead , TA Bailey , AC Woodman
Cranfield Biomedical Centre, Cranfield University, Silsoe, United Kingdom

Lung cancer remains the number one cause of cancer death in the United Kingdom. Genetic abnormalities in lung cancer are being increasingly recognised, although a multi-stage model similar to that described in other cancers, such as colon, has not yet been defined.
Genomic instability is one mechanism thought to play an important part in cancer progression, specifically in the early stages, even before the appearance of any morphological changes. By means of non-radioactive inter-simple-sequence-repeat PCR (Inter SSR-PCR) we have assessed the extent of genomic abnormalities occurring in the progression of non-small cell lung cancer (NSCLC). Inter-SSR PCR amplifies regions located between repeated sequences and allows the detection of chromosomal alterations found in DNA segments.
DNA was extracted from cell lines, of various types and stages of NSCLC, and from a "normal" blood sample. It was then amplified using the inter-SSR PCR technique described. Band differences between "normal" and neoplastic samples were identified by resolution of PCR products on an 8% non-denaturating polyacrylamide electrophoresis gel, followed by silver staining.
By comparison of fingerprints from different sources, a number of intensity changes could be visualized. These results demonstrate the first step in characterising molecular events, which may be indicative of carcinoma development, which in turn may allow more precise and earlier risk assessment for individual patients, enabling more effective therapy.

### P99

### FINE MAPPING OF GENETIC ALTERATIONS ON CHROMOSOME 6 IN UVEAL MELANOMA

JM Risk , J Nunn , B Damoto , KE Burrows , S Hagan , AGM Scholes , P Hiscott , I Grierson , JK Field
United Kingdom, The University of Liverpool, Liverpool

Uveal melanoma is the commonest primary intraocular malignant tumour in adults and approximately 50% of patients develop fatal metastatic disease which correlates with the observation of monosomy 3 in the tumour. In this study, allelic imbalance on chromosome 6 was investigated using 17-37 microsatellite markers in 103 uveal melanomas and the results correlated with prognosis. Allelic imbalance at one or more polymorphic loci was detected in 51/103 (50%) specimens. Twenty-six tumours (25%) showed allelic imbalance at 6p markers, with a minimal region of 13.5Mb being detected between markers D6S344 and D6S1578 (6p25.3-p23). This was associated with disomy at chromosome 3, lack of epithelioid cells, and improved survival. However, allelic imbalance at 6p markers and monosomy 3 were not mutually exclusive. Thirty-five tumours (34%) showed allelic imbalance at markers on 6q, consisting of partial deletion in 20/35. However, this was not consistently observed at any one region on 6q and was not associated with monosomy 3 or survival. The allelic imbalance observed at 6p markers has thus been localised to allow further, gene-specific, studies to be initiated. The data also suggest that long-term prognosis in cases with monosomy 3 is improved in the simultaneous presence of allelic imbalance at 6p markers.

### P100

### A BROAD PANEL OF SCREENING ASSAYS FOR MUTATION AND METHYLATION ASSESSMENT OF GENES INVOLVED IN THE PATHOLOGICAL DEVELOPMENT AND THERAPEUTIC TREATMENT OF NSCLC: UTILITY FOR EARLY DETECTION AND PATIENT MONITORING

SL Lilleberg , C Sanders , J Durocher , M Geimer , C Zhi , KB Walters, G Hong
United Kingdom, Transgenomic, Omaha

Non-small cell lung cancers (NSCLC) contain numerous alterations, including somatic mutations and CpG island hypermethylation in a variety of genes that contribute to the pathological phenotype. Some of these tumor-associated variations have also been detected in the DNA found in the plasma of NSCLC patients, including DNA with mutations in TP53 and KRAS, and hypermethylation in p16INK4a and MGMT. However, a more comprehensive study designed to assess both mutation and methylation status of NCSLC patients at various stages has not been reported. Here we describe a combined assay panel for the assessment of genes associated with disease pathogenesis and therapeutic treatment of NSCLC. DNA from primary tumors and matching patient plasma were screened for mutations in a variety of genes including PTEN, TP53, TP73, KRAS, NRAS, BRAF, MET, EGFR, PDGFRA, PDGFRB, and KIT, and alterations of methylation status in APC, MLH1, p16, p14, MGMT, GSTP1, DAPK, RASSF1A, RUNX3, RARB2, and FHIT. We chose mutation and methylation scanning technology and these targets to develop a thorough screening methodology for alterations known to be associated with NSCLC or that may be involved in resistance to targeted therapeutics. Variations were identified with a denaturing high-performance liquid chromatography (DHPLC) platform that uses post-separation fluorescence technology, enabling the detection of variants that represent < 0.1 – 1.0% of the total analyzed DNA. Using this approach, we identified at least one somatic or epigenetic event in 100% of the NSCLC patients. In no case was a mutation found in the primary tumor that was not also present in the plasma. The results emphasis the heterogenous pattern of genomic alterations and that scanning provides an attractive approach to comprehensive NSCLC genetic and epigenetic screening. The thoroughness of approach may have important implications for screening and staging, and disease monitoring during and following therapy.

### P101

### MICROARRAY ANALYSIS OF OPCML TUMOUR SUPPRESSOR FUNCTION IN THE SKOV-3 OVARIAN CANCER CELL LINE

G.J. Rabiasz [1], D. Scott [1], E.P. Miller [1], E.A. Stronach [2], K.J. Taylor [1], E. Ntougkos [1], H. Gabra [2], J.F. Smyth [1], G.C. Sellar [1]
[1] Cancer Research UK, Edinburgh, United Kingdom, [2] Imperial College, London, United Kingdom

We have identified OPCML (OBCAM) from 11q25 as a tumour suppressor gene frequently inactivated in sporadic epithelial ovarian cancer (EOC), the leading cause of death from gynaecological malignancy. OPCML is a member of the IgLON family of immunoglobulin domain-containing glycosylphosphatidylinositol (GPI)-anchored cell adhesion molecules. GPI-anchored proteins typically localise to lipid raft microdomains in cell membranes, which potentially facilitate rapid signalling into the cell. OPCML transfection into the SKOV-3 ovarian cancer cell lines resulted in phenotypes consistent with tumour suppressor and cell adhesion molecule function: growth suppression *in vitro* and *in vivo*, and enhanced *in vitro* cell-cell aggregation. A somatic mis-sense mutation, P95R, resulted in loss of OPCML-enhanced cell-cell aggregation function, whereas growth suppression remained unaffected. Neither the signalling pathway nor the downstream transcriptional consequences associated with OPCML function have been described. Therefore, we have set out to determine the transcriptional changes associated with OPCML function in SKOV-3 using the CRUK Microarray Consortium 10K cDNA array. We have compared the expression profile of the OPCML-transfected cell line with the SKOV-3 clonal parent cell line, pcDNA3.1zeo-, OPCML antisense-, and OPCML P95R-transfected SKOV-3 cell lines. Expression profiles have been compared using Gene Spring, and genes with >2-fold expression differences identified. In keeping with the phenotypes being analysed, genes with roles in cell adhesion and growth suppression have been identified. Further analysis and validation are in progress and these will be presented.

© 2004 Cancer Research UK

### P102

### GLOBAL PROFILING OF MOLECULAR MECHANISMS OF 5-FLUOROURACIL RESISTANCE IN HUMAN CANCER CELLS

W Wang [1], J Cassidy [2], E Collie-Duguid[3]
[1] Beatson Institute for Cancer Research, Glasgow, United Kingdom,
[2] Beatson Institute for Cancer Research, Glasgow, United Kingdom,
[3] University of Aberdeen, Aberdeen, United Kingdom

The expression of over 22,000 human transcripts were analyzed on Affymetrix HG-U133A oligonucleotide microarrays in 5-pairs of 5-FU resistant and relevant parental cell lines.
In unsupervised hierarchical clustering, 47 probability (Welch $p<0.05$, Benjamini and Hochberg FDR) and threshold ($\geq 1.5$) filtered genes segregated cells into 5FU sensitive and resistant groups. The expression of these genes was not significantly correlated with the primary tumour type from which the cell lines were derived (rectal vs breast). A rule that predicted 5FU sensitivity phenotype with 100% accuracy and high predictive strength (p ratio <0.08) using leave-one-out cross-validation with the KNN algorithm, was built from the expression levels of a subset of 12 of these genes.
In supervised analysis, key pathways reported or hypothesized to be important in the action of 5FU were evaluated. The expression of 101 transcripts was altered in at least 4 of the resistant lines and was > 1.5-fold statistically significantly altered ($p<0.05$) between the resistant and sensitive groups. Protein levels of some genes were verified by Western blot. Expression of a number of genes involved in 5-FU activation were significantly down regulated (TK, 2.9-fold; OPRT, 2.3-fold; UMPK, 3.2-fold; PNP, 3.6-fold) in resistant cells. In line with previous reports, TS and c-yes over-expression were detected. Although NF-κB p65 mRNA over-expression did not reach statistical significance, higher NF-κB DNA binding and transcriptional activities were consistently detected in resistant cells. 5FU resistant cell lines manifested reduced expression of genes governing DNA replication (DNA polymerase α, MCM7), G1-S (cyclin D3, Cdk-2) and S phase (cyclin A) transition. These findings were highly consistent with the slower growth rates of resistant cells and the higher proportion or resistant cells arrested in G1 or early S phase. This phenotype may provide resistant cells with time to repair genetic damage and escape 5-FU induced apoptosis.

### P103

### ARSENIC TRIOXIDE INDUCES CHANGES IN THE GENE EXPRESSION PROFILE OF ACUTE PROMYELOCYTIC LEUKAEMIA AND MULTIPLE MYELOMA CELL LINES

NM Gebril , A Gilkes , K Mills , A Tonks , AK Burnett
University of Wales College of Medicine, Department of Haematology, Cardiff, United Kingdom

Arsenic trioxide ($AS_2O_3$) has recently been used for the treatment of acute promyelocytic leukaemia (APL). The mechanism of action of this drug is thought to involve the induction of apoptosis and partial differentiation. $AS_2O_3$ also exhibits anti-tumour activity on other malignancies such as Multiple Myeloma (MM). The aims of this study were to investigate the effect of $AS_2O_3$ on the gene expression profile of APL & MM cells.
APL (NB4 & NB4R2) and MM (SKO-007 & MC/CAR-Z2) cells were treated with 2 μM $AS_2O_3$ for 24 hours; a dose which demonstrates a significant effect on cell proliferation and clinically achievable. cDNA Microarray analysis (Affymetrix system) was performed to measure and compare the expression levels of genes in control (untreated) and $AS_2O_3$-treated cells.
Data analysis of NB4, NB4R2, SKO-007 and MC/CAR-Z2 cells demonstrated that 3575, 4820, 3775 and 4459 genes, respectively were up- or down-regulated by two-fold in $AS_2O_3$-treated cells. Telomerase expression was down-regulated in all $AS_2O_3$-treated cells, with the most marked effect seen in NB4R2 cells (3.3 to 0.3 fold). $AS_2O_3$ down-regulated all Ras members in APL cells. In contrast, changes in MM cells were varying, K-Ras was up-regulated and H-Ras was down-regulated. Moreover, the *bcl*-2 gene was down-regulated in all $AS_2O_3$-treated cells except in MC/CAR-Z2 MM cells. The major change in the level of *bcl-2* was observed in NB4R2 cells falling from 1.02 to 0.4 fold. $AS_2O_3$ also up-regulated many genes such as cyclin D1, with the least effect observed in NB4R2 cells (0.9 to 1.02 fold). This data might explain the prominent effect of $AS_2O_3$ on refractory/relapsing APL.
In summary, this data demonstrates that $AS_2O_3$ has considerable effects on a wide range of genes involved in many mechanisms such as proliferation and differentiation. In addition, $AS_2O_3$ is more effective in the treatment of refractory APL than ATRA-sensitive APL and MM.

### P104

### HIGH LEVEL EXPRESSION OF GN6ST OBSERVED IN A SUBGROUP OF ACUTE LYMPHOBLASTIC LEUKAEMIA

G Timson [1], S Banavali [2], MI Gutierrez [1], I Magrath [3], KG Bhatia [1], MH Goyns [1]
[1] King Faisal Specialist Hospital, Riyadh, Saudi Arabia,
[2] Tata Memorial Hospital, Mumbai, India, [3] INCTR, Brussels, Belgium

Microarray analysis is a powerful technology, but its main impact on routine diagnosis for the near future may be in revealing individual genes, which are useful diagnostic markers. Recently microarray analysis has identified a novel subgroup of childhood precursor-B acute lymphoblastic leukaemia (ALL) from a unique gene expression profile of over 30 genes (Yeoh et al., 2002, Cancer Cell, 1, 133-143). We have evaluated the four most highly expressed genes from this profile to determine whether any of these genes by itself could be useful as a diagnostic indicator.
The levels of expression of N-acetylglucosamine-6-O-sulfotransferase (GN6ST), protein tyrosine phosphatase receptor M (PTPRμ), G protein-coupled receptor 49 (HG38) and KIAA1099 protein were determined by quantitative real time RT-PCR and quantified by comparison to the level of expression of GAPDH in the same samples. The peripheral blood leukocyte or bone marrow samples studied were from a cohort of 116 pediatric Indian patients diagnosed with precursor-B ALL. In nine cases, GN6ST was over-expressed at levels 15 to 120 times higher than the average of all other samples. PTPRμ was also over-expressed in seven of these nine cases and KIAA1099 in eight of them. HG38 exhibited little correlation with GN6ST expression. The patients investigated in this study were first diagnosed from nine to 18 months prior to the molecular analysis. Although all of the patients exhibiting high level GN6ST expression were alive at this time, compared to 72% of the other patients for whom we had data (60 patients), it is too early to make definitive judgments about the predictive value of GN6ST over-expression.
We suggest that high level expression of GN6ST may prove to be a useful diagnostic marker for a subgroup of previously unclassified ALL.

### P105

### ON THE GENESIS OF BARRETT'S OESOPHAGUS: A THREE DIMENSIONAL STUDY OF THE RELATIONSHIP BETWEEN OESOPHAGEAL GLAND DUCTS, BARRETT'S OESOPHAGUS AND ASSOCIATED SQUAMOUS ISLANDS

RA Coad , PJ Warner , H Barr , NA Shepherd , AC Woodman
[1] Cranfield Biomedical Centre, Cranfield University, Silsoe, United Kingdom, [2] Cranfield Postgraduate Medical School in Gloucestershire, Gloucester, United Kingdom

Barrett's oesophagus, an acquired metaplastic condition of the distal oesophagus, occurs in approximately 10% of patients with gastro-oesophageal reflux disease. The presence of Barrett's metaplasia increases a patients risk of developing adenocarcinoma by 30-40%. This is the cancer with the most rapidly increasing incidence in the western world and the prognosis for patients is poor. Regression of Barrett's oesophagus has been seen in the formation of microscopic and macroscopic squamous islands following various treatment regimes The location of the pluripotential stem cells, which give rise to Barrett's metaplasia and associated squamous islands, is an area of considerable contention. There is some evidence which suggests the oesophageal gland ducts contain this stem cell population, although this is mainly derived from animal models.
We have isolated human oesophageal gland ducts underlying both Barrett's oesophagus and squamous islands. By taking serial sections through these structures we have assessed their interrelationship in the three dimensions. We have found that oesophageal gland ducts show definite morphological continuity with Barrett's metaplasia. This is demonstrated by a gradual transition of the morphological features from the normal cuboidal cells of the duct into the metaplastic cells as the duct opens onto the mucosal surface. This definite interrelationship between the structures has not previously been demonstrated in such a methodical way. We also report the universal association between squamous islands and oesophageal ducts. This finding confirms previous assumption and is of fundamental importance if we are to ascertain the origin of Barrett's oesophagus and develop more targeted treatment strategies.

© 2004 Cancer Research UK

### P106

### THE 825C>T POLYMORPHISM OF THE G-PROTEIN BETA-3 SUBUNIT GENE (GNB3) AND BREAST CANCER

P Krippl , U Langsenlehner , H Samonigg
Division of Oncology, Medical University Graz, Graz, Austria

The 825C>T polymorphism in the gene for the G-protein 3 subunit (GNB3) has been linked to the occurrence of a splice variant of GNB3 and distinct cellular and metabolic features and may be associated with malignant disease. 500 patients with histologically confirmed breast cancer and 500 female age-matched healthy control subjects were genotyped for the GNB3 polymorphism to analyze its role for breast cancer. Prevalences of GNB3 CC, CT and TT genotypes were similar among patients (49.7, 39.8, 10.5%) and controls (50.1, 42.4, 7.5%, $P$=0.25). The GNB3 genotype was furthermore not linked to tumor size, histological grading, estrogen or progesterone receptor status and age at diagnosis. In an exploratory analysis, carriage of a 825-T allele was associated with a longer metastasis-free period in patients with primary low-grade breast cancer, but not in those with primary high-grade breast cancer (Cox regression, $P$=0.025). We conclude that the GNB3 825C>T polymorphism does not appear to be associated with breast cancer risk, but may influence development of metastasis in low-grade tumors.

### P107

### CHARACTERISATION OF NOVEL, FRAGMENT-DERIVED INHIBITORS OF CYCLIN DEPENDENT KINASES 1 AND 2 AS POTENTIAL DRUG-LIKE MOLECULES

JE Curry , R Anthony , V Berdini , DJ Davis , EJ Lewis , L Magor , M O'Reilly , M S Squires , A Woodhead , P G Wyatt
Astex Technology, Cambridge, United Kingdom

Cyclin Dependent Kinases (CDKs) play key roles in regulating the eukaryotic cell cycle. Two of particular interest are CDKs1 and 2, which act at the G2/M DNA damage and G1/S cell cycle checkpoints respectively. Deregulation of the cell cycle is associated with a number of human cancers. As such these kinases are attractive oncology therapeutic targets for small molecule inhibitors in order to modulate these key pathways.
We have used high throughput X-ray crystallography to identify a range of potent, novel fragment based hits against CDK1 and 2. Further rounds of chemical rational design, guided by crystal structures and potency against relevant CDKs, rapidly developed these initial hits into tractable lead molecules with sub-nanomolar potencies against CDKs 1 and 2.
These lead molecules were characterised in a range of cell-based assays that demonstrated their effectiveness as inhibitors of cell proliferation resulting from a specific cell cycle arrest. The mechanism of action of this inhibition was confirmed by monitoring the phosphorylation of downstream substrates.
Furthermore the compounds were shown to exhibit negligible toxicity towards non-proliferating fibroblast cells, and were equipotent in cells overexpressing P-glycoprotein or those lacking p53. Members of active series had good pharmacokinetics.
In conclusion the characterisation of these inhibitors indicates they have drug-like properties, suitable for further development.

### P107:1

### ENGINEERING AUTOLOGOUS MACROPHAGES TO RECOGNISE AND ERADICATE ESTABLISHED TUMOURS

A Biglari , DG Gilham , LB Woolford , LJ Fairbairn
United Kingdom, Paterson Institute for Cancer Research, Manchester

Macrophages extensively infiltrate tumours and can mediate anti-tumour immunity through antigen presentation and cytokine secretion. Importantly, these cells also initiate antibody-dependent cellular cytotoxicity (ADCC) through the activity of Fc-γ-receptors. We aim to exploit these activities through the specific targeting of macrophages to tumour by the expression of chimeric Fc-γ-receptors. The chimeric receptors are targeted by single chain Fvs (scFvs), which specifically bind either carcinoembryonic antigen (CEA) or 5T4 oncofetal antigen fused to the Fc–γ receptor chain. Our present studies have used two differing approaches in order to investigate the anti-tumour activity of modified macrophages in murine model systems. In the first approach, we are assessing the potential of retrovirally modified murine hematopoietic stem cells to generate macrophage cells expressing the recombinant Fc-γ-receptors in order to target antigen-specific tumour cells. In the second approach, we are evaluating the efficacy of adenovirally modified primary human monocytes expressing the Fc-γ-receptors to recognise and kill tumour cells. We have demonstrated that both human and mouse primary monocytes expressing the recombinant Fc-γ-receptors can target and kill tumour cells *in vitro* and, on co-administration with tumour to mice, lead to reduced tumour growth *in vivo* and a survival advantage in these animals. These data suggest that targeted macrophages may be a useful means of delivering a specific anti-tumour effect.

### P107:2

### ABERRANT PROTEIN EXPRESSION OF NEK2 KINASE IN HUMAN TUMOURS

D.G. Hayward [1], R.B. Clarke [2], A.J. Faragher [3], M.R. Pillai [4], I.M. Hagan [2], A.M. Fry [1]
[1] Japan, University of Leicester, Leicester, [2] United Kingdom, Paterson Institute for Cancer Research, Manchester, [3] United Kingdom, MRC Toxicology Unit, Leicester, [4] United Kingdom, Institute of Animal Health, Pirbright

Nek2 is a ubiquitously expressed serine/threonine kinase involved in regulation of the cell cycle. It localises to the centrosome throughout the cell cycle and functional studies in human, *Xenopus*, *Drosophila* and fungal systems implicate it in regulating both centrosome structure and mitotic progression (1). Currently there is considerable interest in determining whether centrosomal protein kinases that promote mitotic progression, such as Cdk1, Aurora A, Plk1 and Nek2, are tumorogenic when upregulated and hence are viable chemotherapeutic targets (2,3).
We report the elevation of Nek2 protein levels both in several cancer derived cell lines and in primary human breast tumours. We demonstrate that Nek2 exhibits increased expression at the protein level in specific prostate, ovarian and leukemic cell lines. Additionally, examination of breast tumours from 20 patients showed protein upregulation in 75% of cases, the first determination of aberrant
Nek2 expression in solid tumours. Finally we show that overexpressing wild type Nek2 in a normal cultured breast epithelial cell background leads to centrosome and nuclear abnormalities indictative of the abnormal segregation of chromosomes
Nek2 is now not only implicated in mitotic defects in cell culture models but also detectable in human tumours from the earliest stages of formation. This presents the possibility of Nek2 acting not only as a prognostic marker but as a viable target for therapeutic intervention.

References

(1) Fry AM. (2002). *Oncogene*, 21, 6184-6194.
(2) Zhou H, Kuang J, Zhong L, Wen-lin K, Gray JW, Sahin A and Brinkley R. (1998). *Nat. Genet.*, *20*, 189-193.
(3) Smith MR, Wilson ML, Hamanaka R, Chase D, Kung H, Longo DL and Ferris DK. (1997). *Biochem. Biophys. Res. Comm.*234, 397-

© 2004 Cancer Research UK

### P107:3

**CO-ORDINATED REGULATION OF THE TUMOUR SUPPRESSOR GENE P16 BY Β-CATENIN-LEF-1 AND ETS TRANSCRIPTION FACTORS.**

C Murphy , M El-Tanani , FC Campbell
United Kingdom, Surgery, Cancer Centre, The Queen's University of Belfast, Belfast

**Background:** Dysregulation of p16 is a crucial step in malignancy. p16 acts as a tumour suppressor and encodes a cyclin dependent kinase inhibitor which disrupts progression of the cell cycle. The p16 promoter sequence contains 2 DNA binding sites for Ets transcription factors and 4 for Lef-1. β-catenin-Lef-1 is central to colorectal tumourigenesis and Ets transcription factors play a role in initiation of these tumours. Together these molecules may play a role in dysfunction of p16 leading to neoplastic transformation in the colon.
**Aims:** This study tests the hypothesis that Ets transcription factors co-operate with β-catenin-Lef-1 in the regulation of p16 transcription.
**Methods:** Rama37 cells were transiently co-transfected with a p16 promoter luciferase reporter construct and a combination of stable mutant β-catenin, Lef-1, Ets-1 and Ets-2 cDNA. p16 promoter transactivation was assessed using a luciferase assay.
**Results:** Over-expression of the stable mutant form of β-catenin or Lef-1 induced p16 promoter reporter construct by a 4-5-fold increase compared to empty vector (control). Co-transfection of expression vectors for Ets-1 or Ets-2 with β-catenin and Lef-1 activated the p16 promoter reporter gene by a 26-fold and 45-fold increase, respectively. PEA3 reduced p16 promoter activity of Ets 1 and β-catenin-Lef-1 to 4 fold.
**Conclusions:** Our data show that Ets-1 and Ets-2 transcription factors synergize with β-catenin-Lef-1 to upregulate p16 transcription whilst PEA3 suppresses p16 promoter activity and may act as a negative competitor of Ets1 and Ets2. These events may contribute to colonic tumourigenesis.

### P107:4

**THE INTERACTION OF TCF-4 ON OSTEOPONTIN GENE EXPRESSION IN RELATION TO BREAST CANCER METASTASIS.**

C Mason , FC Campbell , M El-Tanani
United Kingdom, Surgery, Cancer Centre, The Queen's University of Belfast, Belfast

**Introduction:** Metastasis of common solid tumours is a complex process of both genetic and phenotypic change resulting in tumour cell dissemination. The adhesive glycoprotein osteopontin (OPN) has been implicated not only in disease progression and metastasis in rodents, but also as a prognostic factor of metastasis and patient demise in human cancer. Osteopontin expression is regulated by the Wnt/Tcf signalling pathway. It has been shown that transient transfection of Tcf-4 cDNA reduces OPN levels in rat mammary carcinoma cells, potentially reducing the metastatic ability of these cells. This study will attempt to elucidate the role of Tcf-4 signal transduction in osteopontin regulated metastasis.
**Aim:** To study the mechanism of action of Tcf-4 and other unknown genes upon osteopontin transcription and metastases.
**Materials and Methods:** The effects of Tcf-4 and OPN were investigated *in vitro*. Osteopontin cDNA was cloned into a tetracycline inducible expression system (T-Rex) (Invitrogen), a constitutive pBK-CMV expression vector (Stratagene), each construct was stably transfected into Rama 37 cells. OPN levels were assessed by western blotting. Invasion and migration assays were carried out in matrigel models.
**Results:** The expression of OPN in TRex (– tetracycline) was similar to the control non transfected Rama 37 cells but with 1.2 μg/ml tetracycline was enhanced 6 fold. The constitutive active system pBK-CMV-Rama 37 enhanced 16 fold increased above the non-transfected Rama 37 cells. Over-expression of OPN in TRex system or constitutive expression cells shown to induced invasion and migration ability of Rama 37 cells *in vitro.*
**Conclusions:** The metastatic ability of osteopontin has been confirmed *in vitro*. The effect of Tcf-4 on OPN expression, invasion and migration will be determined.

### P107:5

**REGULATION AND EXPRESSION OF ARGININOSUCCINATE SYNTHETASE IN HUMAN OVARIAN CANCER**

PW Szlosarek [1], MJ Grimshaw [2], GD Wilbanks [3], F Burke [1], JL Wilson [1], FR Balkwill [1]
[1] United Kingdom, Barts and The London, London, [2] United Kingdom, Guy's Hospital, London, [3] United Kingdom, University of South Florida, Tampa

Tumour necrosis factor-α (TNF-α) is implicated in the development of human ovarian cancer, with increased expression linked to the upregulation of proinflammatory pathways, including cytokines, cell adhesion molecules and enzymes. We have recently identified argininosuccinate synthetase (ASS) as a novel proinflammatory enzyme regulated by TNF-α in a subset of ovarian carcinoma cell lines. Here, we extended this observation to a panel of primary ovarian tumour cells, confirming induction by 24hrs following incubation with 20ng/ml of TNF-α. Autocrine TNF-α was required for maximal ASS induction. In contrast, there was no evidence for ASS induction by TNF-α in normal ovarian surface epithelium (OSE). A cancer profiling array revealed increased expression of both TNF-α and ASS mRNA in ovarian tumours compared with corresponding normal tissue. Immunohistochemistry confirmed presence of ASS protein in ovarian cancers with absent staining in normal OSE. We also determined the effect of TNF-α on ASS enzymic activity and showed that TNF-α acts as a survival factor for ovarian cancer cells under arginine-free conditions. Finally, we assessed ASS expression in two murine cancer models and explored the effect of targeting ASS with TNF-α neutralising antibodies on tumour development. In summary, we show that ASS, with its dual role in nitric oxide synthesis and the arginine-citrulline cycle, is upregulated in human ovarian cancer. Moreover, ASS expression could be modulated using specific TNF-α neutralising antibodies and the combination with arginine depletion may provide a novel approach to the treatment of ovarian cancer.

### P107:6

**EXPRESSION PROFILING OF CHROMATIN REMODELLING GENES IN BLADDER AND RENAL CANCERS**

A Veerakumarasivam , H Ozdag , A Terschendorff , S Batley , I Mills , D Neal , T Kouzarides , C Caldas , J Kelly
United Kingdom, University of Cambridge, Cambridge

Post translational modification of histones that are involved in chromosome remodelling appears to have a key role in the regulation of gene expression. Transcription becomes active when histones are acetylated by histone acetyltransferases (HAT), silenced when histones are deacytelated by histone deactylases and silenced or activated when methylated by histone methyltransferases (HMT). However, deacetylase activity can contribute to gene activation as well, as shown previously in *Drosophila* and *Saccharomyces cerevisiae*. Acetylation of core histones has also been correlated with cellular processes, including chromatin assembly, DNA repair, and recombination. The combination of these modifications gives rise to what is known as the "histone code" which could result in epigenetic changes in tumorigenic pathways. Expression of Histone Deacytelases (*HDAC1, HDAC2, HDAC4, HDAC5, HDAC7, hSIRT1*), Histone Methyltyransferases (*SUV39H1, SUV39H2*) and Histone Acetyltransferases (*P300, CBP, PCAF*) was investigated using real-time QRT-PCR on 127 bladder tumours, 20 normal bladder, 80 renal tumours, 12 normal renals and 7 bladder cell lines (UMUC-3, EJ28, HT1197, HT1376, RT112, T24 and 253J). This study on chromatin remodelling genes will give important insights into the tumorigenesis and the molecular profiling of bladder and renal tumours.

© 2004 Cancer Research UK

### P108

### NOVEL ANTICANCER PROPERTIES OF POMEGRANATE EXTRACTS; SUPPRESSION OF PROLIFERATION, INVASION AND XENOGRAFT GROWTH OF HUMAN PROSTATE CANCER CELLS

L.M. Gommersall [1], M. Albrecht [2], W.G. Jiang [3], J. Kumi-Diaka [5], E.P. Lansky [4], M.J. Campbell [1]
[1] Institute of Biomedical Research, University of Birmingham, Birmingham, United Kingdom, [2] Institute of Anatomy, Philipps University, Marburg, Germany, [3] Department of Surgery, University of Wales College of Medicine, Cardiff, United Kingdom, [4] Rimonest Ltd., Nesher, Israel, [5] Divisionof Science, Florida Atlantic University, Daivie, United States

We have investigated the effects of several Pomegranate fruit (*Punica granatum)* extracts on the proliferation of normal, benign hypertrophic, and malignant prostate cells. Pomegranate juice contains anthocyanins and phenolic acids with demonstrated antioxidant and anticancer properties. Cell proliferation and cell cycle analyses combined with staining of apoptotic cells were used to determine the effects of cold-pressed seed oil (*Oil*), fermented juice polyphenol fractions (*W*) and aqueous pericarp extract (*P*) on prostate cells. To evaluate changes in the gene expression profile following extract stimulation, we performed real-time RT-PCR studies. *In vitro* cell invasion assays and xenograft experiments were performed to test the influence of pomegranate extracts on cell invasion and metastasis.

*Oil*, *W* and *P* all acutely inhibited *in vitro* proliferation of LNCaP, PC-3 and DU 145 cancer cell lines. The dose of P required to inhibit cell proliferation of the androgen independent prostate cancer cell line DU 145 by 50% ($ED_{50}$) was 30 μg/ml, whereas normal prostate epithelial cells (hPrEC) were significantly less affected ($ED_{50}$ 250 μg/ml). These effects were mediated by changes in cell cycle distribution and induction of apoptosis. For example, DU 145 cells showed significant increase from 11% to 22% in $G_2$/M cells ($p<0.05$) by treatment with *Oil* (35 μg/ml), and induction of apoptosis. These cellular effects coincided with rapid changes in mRNA levels of gene targets. Thus, 4 hr treatment of DU 145 cells with *Oil* (35 μg/ml) resulted in significant 2.3 fold upregulation of the CDKI $p21^{(waf1/cip1)}$ ($p<0.01$) and 0.63 fold downregulation of c-*myc* ($p<0.05$). In parallel, all agents potently suppressed PC-3 invasion through Matrigel, and furthermore *P* and supercritical $CO_2$ extracted pomegranate seed oil (*S*) demonstrated potent inhibition of PC-3 xenograft growth in athymic mice. Overall this study demonstrates significant antitumor activity of pomegranate-derived materials against human prostate cancer.

### P109

### IDENTIFICATION OF STANDARD REFERENCE COMPOUNDS FOR USE IN A PANEL OF HUMAN XENOGRAFTS IN NUDE MICE

PT Anstruther [1], S Lynagh [1], S Crawford [1], J McPhail [1], J Gillard [1], R Hull [2], JA McKay [1]
[1] Quintiles Limited, Edinburgh, United Kingdom, [2] James Black Foundation, London, United Kingdom

The nude mouse xenograft model is a well-established technique used to study the anti-tumour activity of potential novel therapeutics. However, available data detailing specific interactions between reference compounds and tumour cell lines is limited. Hence, we have tested a number of utilised chemotherapeutics against a range of human tumour xenografts in the nude mouse.

Cell suspensions were grown *in vitro,* and implanted subcutaneously into the flank of athymic nude mice. Once palpable tumours had established, mice were treated with various anti-cancer drugs, and tumour volumes measured regularly.

Paclitaxel (20 mg/kg i.v.) significantly reduced growth of the uterine xenograft MES-SA ($P<0.001$), and the colorectal tumours HT-29, HCT116 and LoVo ($P<0.01$), however no significant reduction in growth rate was observed in the colorectal xenograft DLD-1. Gemcitabine (Gemzar®, 100 mg/kg i.p.) significantly reduced the tumour volume of BxPC-3 (pancreatic) and LoVo xenografts ($P<0.001$). The growth of LoVo tumours was also reduced following treatment with 5-fluorouracil (5-FU, 50 mg/kg i.v.; $P<0.05$). However, no significant effect on tumour growth was observed in DLD-1 or HCT116 xenografts when treated with 5-FU (25 mg/kg i.v.). Mitomycin C (2 mg/kg i.p.) significantly reduced the mean tumour volume of the NCI-H460 lung xenograft ($P<0.001$), but was without influence on HT-29 or MES-SA tumours. Cisplatin (4 mg/kg i.v.) significantly reduced mean tumour volume of the NCI-H460 xenografts ($P<0.01$), while no significant reduction in growth rate was observed in MES-SA tumours.

In conclusion, variable efficacy of chemotherapeutics was observed between human tumour xenografts. This data is pertinent for the selection of appropriate reference compounds for evaluation of novel anti-cancer compounds *in vivo*.

### P110

### FEASIBILITY OF UTILISING THE HOLLOW FIBRE (HF) ASSAY TO INVESTIGATE THERAPEUTIC RESPONSE IN THE EWING'S SARCOMA FAMILY OF TUMOURS (ESFT)

EM Bridges [1], MC Bibby [2], S Dalal [1], SA Burchill [1]
[1] Cancer Research UK, St James's University Hospital, Leeds, United Kingdom, [2] TCCRC, Bradford University, Bradford, United Kingdom

The NCI HF anti-cancer drug screen is a rapid *in vivo* assay to evaluate novel chemotherapeutic drugs prior to full xenograft screening. The feasibility of using HF to evaluate the effects of chemotherapeutic drugs and novel treatment strategies in ESFT cells has been assessed. Viable cell number and growth kinetics of six ESFT cell lines in HF has been studied by the trypan blue exclusion assay. The histology of the ESFT cells in paraffin-embedded HF has been examined by staining with H&E and microscopy. Assays for proliferation (immunohistochemistry for Ki67) and apoptosis (TUNEL) have been optimised for use in the HF. The effects of doxorubicin (0.1μg/ml) on cell morphology, proliferation and apoptotic index of ESFT cells seeded in HF have been evaluated. All six ESFT cell lines have been successfully seeded into HF. Viable cell number increased exponentially with time (0-96h). A673 cells grew as spheroids with a highly proliferative outer rim (100μM) and an inner necrotic centre. SK-N-MC cells formed cell clumps, and the remaining cell lines studied grew as a homogenous mass within the HF. Two cell lines, RD-ES and SK-ES1, produced rosettes consistent with ESFT histology. Following treatment with doxorubicin viable cell number within the HF decreased in a time dependent manner (24-96h). The A673 cells in HF were more resistant to the induction of apoptosis by doxorubicin than the other cell lines studied, consistent with effects on substrate adherent cultures. Doxorubicin increased the apoptotic index of cells in HF; this was associated with a decrease in proliferation. The organised morphology of A673 spheroids was lost in a time dependent manner after exposure to doxorubicin. In conclusion the HF assay may provide a reproducible high through-put screen to assess novel therapeutic strategies in ESFT. Using the HF it is possible to obtain pure populations of ESFT cells to evaluate tumour markers and biologically significant end-points in a pharmacologically relevant *in vivo* model.

### P111

### A QUANTITATIVE METHOD FOR MEASURING THREE-DIMENSIONAL INVASION IN VITRO USING ORGANOTYPIC CULTURES

M Nystrom [1], M Stone [2], GJ Thomas [3], I Mackenzie [4], IR Hart [1], JF Marshall [1]
[1] Cancer Research UK Clinical Centre, London, United Kingdom, [2] Richard Dimbleby Department of Cancer Research, London, United Kingdom, [3] Eastman Dental Institute, London, United Kingdom, [4] Bart's and the London Medical and Dental School, London, United Kingdom

Invasion by carcinoma cells requires an active interplay with cells of the host stroma, particularly the fibroblasts. Thus assays that measure tumour invasion in vitro in the presence of fibrobasts better replicate the in vivo situation. In order to study invasion in vitro we have used an organotypic skin model, a technique whereby a stratified epithelium is created by plating carcinoma cells onto a collagen gel embedded with fibroblasts. Although invasion through the gel looks similar to the in vivo situation a major disadvantage is that this method is not quantitative and thus has limited use as a tool for assessing and comparing the efficacy of anti-invasive therapies. To address this problem we have devised a computer-based method of quantitating invasion. Sections (4um) cut from paraffin embedded gels were stained with the pan-cytokeratin antibody AE1/AE3 and were photographed. Using image analysis software (Optilab;Graftek, France) a calibrated binary image was generated which then was converted into a 'convex' image that encompassed all of the carcinoma tissue. From this single image the mean chord Y (mean vertical depth of invasion) was calculated. Image analysis of serial sections showed that carcinoma invasion varied only 5-10% across 180um of the centre of gels confirming that the assay was robust. Using this method we have shown that human foreskin fibroblasts promote five-fold more invasion of CA1 cells (an oral squamous cell carcinoma line) than do NIH 3T3 murine fibroblasts confirming that the source of fibroblasts has an important impact on tumour cell behaviour. Various inhibitors of invasion have been screened and it was shown in several replicate experiments that the cyclo-oxygenase inhibitor, NS398, inhibits invasion of CA1 cells by 50% . We propose that this method for quantitating invasion in the organotypic skin model is a valuable, reliable and accurate way of screening for anti-invasive therapies.

© 2004 Cancer Research UK

### P112

### USE OF A PRIMARY LYMPHOMA CULTURE SYSTEM TO INVESTIGATE THE *IN VITRO* ACTIVITY OF BORTEZOMIB

L Maharaj [1], SJ Strauss [1], J Stec [2], SP Joel [1], TA Lister [1]
[1] Department of Medical Oncology, St. Bartholomew's Hospital, West Smithfield, London, United Kingdom, [2] Millennium Pharmaceuticals, Boston, United States

A primary lymphoma culture system based on the CD40-CD40L interaction has previously been reported (Johnson et al, Blood. 1993 5;82(6):1848). This has been modified and validated prior to evaluating the activity of the proteasome inhibitor bortezomib in primary follicular lymphoma (FL) and mantle cell lymphoma (MCL) cultures.

Single B-cell suspensions were isolated from lymph nodes, ascites or peripheral blood from 10 patients with histologically confirmed FL or MCL. After disaggregation and/or density gradient separation, cells were plated at a density of $5x10^5$ cells/well into 96-well plates containing CHO cells transfected with the CDw32 Fc receptor to express the CD40 ligand, which, when irradiated to prevent further proliferation, provide a stromal layer supporting B-cell proliferation. Malignant or normal B-cells were cultured in IMDM medium supplemented with 10% human serum and 2ng/ml IL-4, and could be maintained for up to 8 days whilst retaining close phenotypic resemblance to the original sample, confirmed by flow cytometric quantitation of cell surface markers. After 24hrs drug was added at varying concentrations in triplicate and cell number and viability were assessed after a further 48hrs using the trypan blue exclusion assay. $EC_{50}$ values for % viability were derived for each sample using Graphpad Prism.

MCL cells were typically more sensitive to bortezomib than FL cells (median $EC_{50}$ 156ηM range (123-727ηM), vs 925ηM (206-1600ηM) respectively, Mann-Whitney U-test *P*=0.09, n=5 for each). In contrast, there was no difference in sensitivity to doxorubicin between MCL and FL cells (median $EC_{50}$ 4.1μM range (2.0-6.3μM) vs 2.3μM (1.5-5.5μM)), these patients having previously failed an anthracycline-containing regimen. Furthermore, three of the MCL samples were from patients subsequently treated with bortezomib. *In vitro* sensitivity to the drug correlated with clinical response, suggesting the possible use of this system as an *in vitro* predictor of response to chemotherapy.

### P113

### A DOSE-ESCALATION-STUDY EVALUATING THE TOXICITY AND THERAPEUTIC EFFICACY OF $^{131}$I LABELLED CHIMERIC ANTIBODY CHT-25 IN PATIENTS WITH IL-2 EXPRESSING LYMPHOMAS. PRELIMINARY RESULTS

JA Violet , R Francis , JR Buscombe , J Croasdale , S Parker , P Amlot , AJ Green , A Hilson , RH Begent
United Kingdom, Royal Free Campus, University College London, London

In this phase I/II study $^{131}$I labelled CHT-25 (a chimeric human/ mouse IgG1 whole antibody) directed against the IL2 receptor has been administered to patients with IL2 expressing NHL and Hodgkin's disease. Cohorts of three patients with refractory, relapsed lymphomas were treated with equal doses of CHT-25 labelled with $^{131}$I. Activities were administered from 370 MBq/m$^2$ to 2990 MBq/m$^2$. Patients receiving activities greater than 1440 MBq/m$^2$ required stem cell harvesting. F-18 FDG PET and CT were used to assess response with SPECT and blood activity counting to determine biodistribution. Toxicity was assessed according to NCI toxicity criteria, response by WHO criteria

7 patients have been recruited including 4 with Hodgkin's and 3 with non-Hodgkin's lymphoma. Six of the seven patients have previously undergone high dose chemotherapy with peripheral stem cell rescue. Patients received activities ranging from 488 MBq to 4762 MBq per administration. Four patients had multiple therapies with the maximal administered total activity being 10.7GBq. 2 patients, receiving the lowest activity, discontinued treatment after the first cycle due to progression. One patient has achieved complete remission based upon F-18 FDG PET imaging and CT which is sustained for greater than 12 months. A partial response has been seen in another 2. A further patient achieved stable disease following a single treatment. There has been a single toxic death. Blood activity counts showed persistent circulating antibody with a $T^1/_2$ of more than 50 hours; this is also reflected in the scan appearances up to 96 hours post injection.

In conclusion, this preliminary analysis of $^{131}$I labelled CHT-25 has demonstrated an encouraging response which appears related to administered activity in this heavily pre-treated group of IL2 expressing Hodgkin's disease and NHL. This has been achieved with manageable toxicity and justifies further study.

### P114

### *IN VITRO* PREDICTION OF CYTOCHROME P450 MEDIATED INTERACTIONS BETWEEN CHEMOTHERAPEUTIC DRUGS AND COMMON CO-MEDICATIONS

SJ Brown [1], CJ Ward [1], J Yu [1], M Paine [1], CR Wolf [1], EM Rankin [2]
[1] United Kingdom, Cancer Research UK Molecular Pharmacology Unit, Biomedical Research Centre, Ninewells Hospital and Medical School, Dundee, [2] United Kingdom, Division of Cancer Medicine, Ninewells Hospital and Medical School, Dundee

Cancer chemotherapy is an area of medicine where significant drug interactions are likely to occur. Most chemotherapeutic agents have a narrow therapeutic index and are used in combination with many other drugs that relieve disease symptoms and side effects of treatment. Adverse drug effects caused by co-medications may go unrecognised as they may be assumed to be side effects of the chemotherapy. Conversely, interactions between co-medications and anti-cancer drugs may produce side effects of such severity that subsequent doses of the anti-cancer drug are reduced (potentially compromising therapeutic efficacy) whereas in fact the co-medication should be altered. Interactions between commonly used medications and cytotoxic drugs may arise by inhibition of cytochrome P450 (CYP) enzymes. These interactions could lead to alteration of the circulating concentration of cytotoxic drugs, causing increased toxicity or reduced efficacy. In this study we have evaluated a range of over 40 common medications (identified from an audit of over 100 newly diagnosed cancer patients) for their ability to inhibit three medically important CYP isoforms – CYP2C8, CYP2D6 and CYP3A4, which are involved in the metabolism of many anti-cancer drugs as well as anti-emetics and analgesics. These tests identified a number of commonly used drugs which inhibit CYP isoforms *in vitro* at clinically relevant concentrations e.g. omeprazole (CYP2C8), fluoxetine (CYP2D6) and domperidone (CYP3A4). Drugs identified as potent inhibitors of CYP450s should be considered as potentially being involved in drug-drug interactions and further study *in vivo* to confirm this would allow development of strategies to avoid unwanted interactions with chemotherapy.

### P115

### *IN-VIVO* PHENOTYPING FOR CYTOCHROME P450 CYP3A4 IN CANCER PATIENTS

SJ Brown [1], CJ Ward [1], D Forbes [1], CR Wolf [1], EM Rankin [2]
[1] United Kingdom, Cancer Research UK Molecular Pharmacology Unit, Biomedical Research Centre, Ninewells Hospital and Medical School, Dundee, [2] United Kingdom, Division of Cancer Medicine, Ninewells Hospital and Medical School, Dundee

The drug-metabolising enzyme cytochrome P450 (CYP) 3A4 shows marked variability, which may explain some of the variation in pharmacokinetics of cytotoxic drugs which are metabolised by CYP3A4. In those where the dose of anti-cancer drug (based on Body Surface Area) is too high, excessive toxicity will lead to dose reduction in subsequent treatment cycles. Approximately 30% of patients are thought to be undertreated, which would impact on anti tumour effects of therapy. A reliable means of predicting in which patients the dose needs escalation is required. Assessment of CYP3A4 activity may give a more accurate way of determining the dose of these drugs for an individual patient. To determine an individual's CYP3A4 activity we have used midazolam in an *in-vivo* phenotyping test. We have looked at 30 subjects to see if this method is safe and applicable in cancer patients, who may have liver disfunction and may be taking medications which can induce or inhibit CYP3A4 activity. Cancer patients were given 1mg of intravenous midazolam, and the pharmacokinetic parameters were evaluated by serial blood sampling over 6 hours. 5-fold inter-individual variation was seen in Midazolam AUC which reflects variation in CYP3A activity. In our patients the midazolam concentration at 2 hours correlates with AUC ($r^2$=0.79). We will correlate *in-vivo* phenotyping data for CYP3A4 (using a limited sampling strategy) with levels of cytotoxic drugs which are metabolised by CYP3A4.

© 2004 Cancer Research UK 

### P116

### PHARMACOKINETIC AND PHARMACODYNAMIC EVALUATION OF RH1 DURING A PHASE I CLINICAL TRIAL.

TH Ward [1], S Danson [2], M Dawson [1], H McGrath [3], O Denneny [1], J Cummings [1], C Dive [1], M Ranson [2]
[1] Poland, Clinical and Experimental Pharmacology, Paterson Institute for Cancer Research, Christie Hospital NHS Trust, Wilmslow Road, Manchester, [2] United Kingdom, Department of Medical Oncology, Christie Hospital NHS Trust, Wilmslow Road, Manchester, [3] United Kingdom, Cancer Research UK Drug Development Office, London

RH1 (2,5-diaziridinyl-3-[hydroxymethyl[-6-methyl-1,4-benzoquinone) is a novel bioreductively activated drug which is an excellent substrate for DT-diaphorase (EC. 1.6.99.2). This obligate two-electron reductase diaphorase, which has been shown to be over expressed in many tumours relative to the normal tissue, will reduce RH1 to a hydroquinone producing a powerful DNA cross linking agent. RH1 is presently undergoing a phase I trial. Pharmacokinetic analysis of plasma samples taken on day 1 and day 5 of cycle 1 showed detectable levels of drug with a half life of approximately 6 min for clearance from the blood. The peak levels range from 17 to 113 nM with escalating dose. These dose levels are consistent with those causing significant biological activity *in vitro*. Patient's lymphocytes exposed to RH1 on infusion were analysed using the comet-X assay, which detects DNA interstrand cross links. Briefly cells were collected and irradiated with gamma radiation as were the control pre-infusion lymphocytes. It was expected that interstrand cross-links produced by RH1 would retard the migration of DNA during electrophoresis resulting in less DNA in the tail of the comets compared to the irradiation only controls. To date 5 patients have been treated with RH1 and peripheral blood lymphocytes isolated from pre-infusion and post infusion time points on both day 1 and day 5 of treatment. These were subjected to the comet-X assay and the amount of DNA present in the tail of the comets following irradiation was measured. Along with these samples internal QC samples representing low and medium levels of DNA cross linking were run. The pooled data for all patients on day 1 showed 70-80% DNA in the tail similar in distribution to the irradiated control, however by day 5 the PBLC population showed peaks at 60-70% DNA in the tail suggesting low level cross-linking similar to the low level internal control. Statistical analysis of comets from all patients on day 5 showed a significant difference to those measured on day 1 (p=0.002, T-test).

### P117

### THE ROLE OF TUMOUR VASCULAR MATURATION IN DETERMINING RESPONSE TO TUBULIN-BINDING VASCULAR TARGETING AGENTS

CR Ireson [1], SA Hill [1], G Lewis [1], A Steele [1], C Coralli [1], GU Dachs [1], P Barber [1], D Honess [1], C Kanthou [1], D Shima [2], G Tozer [1]
[1] Gray Cancer Institute, Mount Vernon Hospital, Northwood, Middlesex, HA6 2JR, Northwood, United Kingdom, [2] Eyetech Research Center, 42 Cummings Park, Woburn, MA019801, Woburn, United States

Combretastatin A-4-phosphate (CA-4-P), which is isolated from the South African Bush willow tree *Combretum caffrum*, shuts down blood flow in a range of human tumour xenografts in mice. Furthermore, administration of a single i.v. dose of CA-4-P to patients with advanced solid tumours caused a significant decrease in tumour blood flow. Although the specific tumour characteristics determining susceptibility to CA-4-P have not been established, it may be a consequence of an immature vasculature with relatively little smooth muscle actin (SMA). Fibrosarcoma cell lines, which express only single vascular endothelial growth factor (VEGF) isoforms, were developed from corresponding knock-out mice and grown s.c. in SCID mice. There was no significant difference in the growth rate of w/t tumours and those expressing only $VEGF_{120}$ or $VEGF_{188}$ (doubling time~2.5 days). Tumours were excised, stained for SMA & CD-31 and % of SMA-stained vessels calculated (=vascular maturity index). VMI was 30, 70 & 100% for $VEGF_{120}$, w/t & $VEGF_{188}$ tumours respectively, confirming the importance of the high molecular weight isoform in vascular maturation. Tumours were also grown in dorsal skin flap window chambers in SCID mice for intravital microscopy. Fragments of $VEGF_{120}$ tumours expanded rapidly in the chambers despite poor internal vascularization. Within 4 days of implantation, haemorrhage of the surrounding blood vessels was observed. In contrast, the rate of $VEGF_{188}$ fragment growth was slower until the tumours were well vascularized, with narrower vessels and no haemorrhage. It will be important to determine the vascular response of these tumours to CA-4-P, in order to understand the functional and morphological characteristics that impinge on the responsiveness of tumour blood vessels to vascular targeting agents.
This work was supported by Cancer Research UK.

### P119

### TARGETING NOS EXPRESSION IN HYPOXIC TUMOUR CELLS TO IMPROVE AQ4N DRUG RESPONSE

EC Chinje , Z Kong , S Singh , RL Cowen , KJ Williams , IJ Stratford
United Kingdom, University Of Manchester, Manchester

AQ4N is a novel di-*N*-oxide prodrug topoisomerase II inhibitor currently in Phase I trial in combination with radiotherapy. We have recently shown that up-regulation of inducible-NOS in tumours cells could enhance metabolism of bioreductive prodrugs. NOS is highly expressed in a broad spectrum of human cancers and consists of an oxidase and a reductase domain. The former is a haem-based protein similar to members of the P450 family, whereas the reductase domain shares a high degree of sequence homology with P450 reductase (P450R). Studies to date have revealed a requirement for P450 or the presence of both haem and P450R in the reductive metabolism of AQ4N. The aim of this study was to evaluate the role of human NOS expression in tumours as a potentially exploitable target to improve AQ4N drug response.
We have employed an expression vector containing the cDNA for human iNOS to stably transfect human HT1080 (fibrosarcoma) and MDA231 (breast adenocarcinoma) tumour cells. Two clones have been selected and used in the study: HT-NOS12 (derived from HT1080wt) and MDA-NOS10 (derived from MDA231wt).
**Results**. AQ4N metabolism and enzyme profiling of NOS clones

| Cell line | Rate of metabolism (nM/min/mg )* | | NOS Activity (pmol/min/mg) | | Reductase activity (nmol /min/mg) | |
|---|---|---|---|---|---|---|
| | AQ4 | AQ4M | NOSR | DTD | $b_5R$ | |
| HT1080wt | n.d (2.28) | 6.9 | 0.89 | 3.2 | 244 | 59.4 |
| HT-iNOS12 | 10.3 (72.8) | 130.5 | 95.20 | 24.3 | 262 | 54.2 |
| MDA231wt | n.d (1.47) | 3.9 | 0.67 | 3.5 | 9 | 34.2 |
| MDA-NOS10 | 1.5 (94.9) | 41.3 | 66.22 | 22.1 | 7 | 37.4 |

*Numbers in brackets represent formation rate when AQ4M is used as substrate. n.d. not detectable.

Results from colony formation assays showed that NOS increased sensitivity to AQ4N and at low $O_2$ tension (<1%$O_2$).
In conclusion, our data suggest that the human iNOS enzyme can contribute to the reductive metabolism of AQ4N. We show elsewhere (Simendra *et al.,* this conference) that NO production increases the radiosensitivity of hypoxic tumour cells. Hence, the combination of these two effects provides an opportunity for improving the treatment of radiation- and chemo- resistant hypoxic tumours.

### P120

### SELECTIVE ACTIVATION AND X-LINKING ABILITY OF A QUINONE-MUSTARD CONJUGATE (MUP03/704) IN VITRO : AN NQO1-TARGETED PRODRUG FOR CANCER THERAPY

M Volpato [1], PM Loadman [1], N Abou Zied [2], M Jaffar [2], I Stratford [2], RM Phillips [1]
[1] United Kingdom, Cancer Research Unit, Bradford University, Bradford, [2] United Kingdom, School of pharmacy, Manchester University, Manchester

NQO1 (NAD(P)H: quinone oxidoreductase 1) is a cytosolic flavoprotein that catalyses the two-electron reductions. It is known to bioreductively activate certain quinones and elevated levels of NQO1 activity or mRNA have been observed in human tumours such as NSCLC. NQO1 is therefore a potential target for rational drug development. The purpose of this study is to evaluate a novel class of quinone based compounds whereupon a cytotoxic moiety is released after reduction of the prodrug: the quinone prodrug MUP 03/704 can be bioreductively reduced by NQO1 to give the hydroxyquinone which spontaneously breaks down to afford a non toxic lactone (MUP 03/702) and the alkylating agent (MUP 03/706). In the absence of reduction, the prodrug is potentially non-toxic.
The substrate specificity of MUP 03/704 for NQO1 was measured using spectrophotometry. It exhibited a Km of approximately 940 µM and a Vmax of 5,77 mmol/min/mg. The ability of MUP 03/704 and MUP 03/706, its cytotxic moiety, to induce formation of interstrand crosslinks (ICLs) was evaluated in H460 lung cancer cells (high NQO1 activity) and BE colon cancer cells (no NQO1 activity), using the comet assay. After 1h exposure, 40 µM of MUP 03/704 concentrations, around 40% of DNA crosslinked was measured in H460 cells and only 5% in BE cells. In the same settings, MUP03/706 induced up to 50% in H460 cells and 70% in BE cells. As an example, high concentrations Mitomycin C can induce up to 90% in H460 cells after 1h exposure, and around but less then 10% at its $IC_{50}$.
These results suggest that compound MUP 03/704 could be effectively reduced by NQO1 in cell free system and induced formation of ICLs in cancer cells expressing the enzyme. Further studies are carried out to further characterise its pharmacologic features and to investigate the relationship between its bioactivation and cell lines sensitivity.

© 2004 Cancer Research UK

